# Sex- and gender-related differences in inflammatory bowel diseases

**DOI:** 10.3389/fgstr.2023.1199687

**Published:** 2023-10-03

**Authors:** Irina Blumenstein, Elena Sonnenberg

**Affiliations:** ^1^ Department of Gastroenterology, Hepatology and Clinical Nutrition, University Hospital, Goethe University Frankfurt, Frankfurt, Germany; ^2^ Charité- Universitätsmedizin Berlin, Corporate Member of Freie Univeristät Berlin, Humboldt-Universität Zu Berlin, Berlin Institute of Health, Medical Department of Gastroenterology, Infectious Diseases and Rheumatology, Berlin, Germany

**Keywords:** IBD, gender and sex, treatment reality, psychological impact, treatment differences

## Abstract

This review provides an overview of the current data regarding sex- and gender-specific aspects in patients with inflammatory bowel diseases. A particular focus will be on disease course, medical and surgical treatment strategies, psychosocial differences, and special requirements during pregnancy and family planning. The most significant and clinically meaningful gender differences in IBD relate to psychosocial functioning. Although depression, fatigue, anxiety disorders, eating disorders, and sexual dysfunction also occur in male IBD patients, women seem to be affected much more frequently and severely in these areas.

## Introduction

In inflammatory bowel disease (IBD), sex- and gender-related differences in genetics, epidemiology, disease phenotype, IBD diagnosis, and treatment modalities are not commonly considered (see also **Box 1**). However, when looking at the available studies in detail, there are some gender-specific differences. This review provides an overview of the current data regarding sex- and gender specific aspects in patients suffering from IBD. A particular focus is disease course, medical and surgical treatment strategies, psychosocial differences, and special requirements during pregnancy and family planning. Finally, this review provides suggestions for areas for future research to focus on.

Box 1 **Sex vs. gender (ChatGPT, 2 November 2022. Prompt: explain the difference between sex and gender in health).**

**Sex** refers to a person’s biological and physiological characteristics, including chromosomes, hormones, and reproductive organs. Typically, individuals are classified as male or female based on their anatomy and genetics.

**Gender**, on the other hand, refers to the socially constructed roles, behaviors, activities, and attributes that a given society considers appropriate for men and women. Gender identity refers to an individual’s deeply held sense of their own gender, which may or may not align with their assigned sex at birth.

In the context of health, it is important to consider both sex and gender because they can influence health experiences, access to health care, and health outcomes. For example, women and men may experience different symptoms or have different risk factors for certain health conditions. In addition, societal expectations and norms around gender can affect health behaviors and decisions, such as seeking preventive care or engaging in healthy behaviors.

Recognizing and addressing the influence of both sex and gender can help ensure that health care is inclusive and effective for all individuals.

## Epidemiology and environmental factors

The gender-specific distributions of Crohn’s disease (CD) and ulcerative colitis (UC) depend, among other things, on ethnicity. Although women in Europe and the USA are affected approximately twice as often as men by Crohn’s disease, in Asia more men are affected than women. In UC, there is no difference in incidence related to sex, nor between continents (1–9). A recently published study representing 16 regions in Europe, North America, Australia, and New Zealand revealed an interesting relationship between age and sex in IBD epidemiology: in this study, women had a lower risk of CD in childhood up to the age of 10–14 years (incidence rate ratio 0.70; 95% confidence interval (CI): 0.53 to 0.93). Thereafter, however, a higher risk of CD was seen, which was statistically significant in the age groups 25–29 years and over 35 years. There was no significant difference in the incidence of UC between female and male patients (except for the 5–9 years age group) up to the age of 45 years; thereafter, a significantly higher incidence of UC was observed for male patients than female patients. Overall, men had a 20% higher incidence rate of UC than women from the age of 45 years (10).

When looking for causes, pediatric studies show that the association between antibiotic exposure and the development of IBD was stronger in boys than in girls (11, 12). Gender-specific associations have also been shown for other risk factors, such as appendectomy, which increases the risk of Crohn’s disease in women (13). Similarly, nicotine abuse increases the risk of Crohn's disease in women in particular. (14). Somewhat surprising is the high proportion of women with Crohn’s disease who smoke, which is significantly higher than that of men (15, 16). Smoking is considered to be a protective factor in UC, but a Dutch study showed that this applies only to men. This study also found that only men who smoke have a lower need for immunosuppressive therapy (17). Women, therefore, have the greatest benefit if they stop smoking and should primarily be supported by smoking cessation programs.

Epidemiological studies have shown that the use of hormones such as oral contraceptives (OC) or post-menopausal hormone replacement therapy (HRT) in women is associated with the occurrence of IBD and complicative courses (18). A meta-analysis by Ortizo et al. found that OC users had a 24% and 30% higher risk of developing CD and UC, respectively (19). A large prospective cohort study also found a positive association between the use of hormone replacement therapy and the risk of developing CD (20). Furthermore, the use of combined OC confers a higher risk of CD-related surgery in long-term users (21). No increased risk for the development of IBD can be derived from the current study situation on HRT. Kane et al. (22) even documented a dose-dependent protective effect of HRT on disease severity in IBD. Other environmental factors, such as nutrition, could also play a gender-specific role, but robust data are lacking.

## Genetics

If there is a family history of IBD women are more likely to be affected than their male relatives, this so-called female imprinting is observed, which corresponds to a higher female-to-female transmission rate (23). In addition, some susceptibility gene variants are associated with a modulation of the gender-specific risk for women, including the genes influencing the IL-23 and IL-10 signaling pathways (24, 25). So far, however, these genetic differences have not had any influence on diagnosis, prognosis, or treatment decisions.

## Extraintestinal manifestations

The gender-specific occurrence of extraintestinal manifestations is well established (26–31). The best-known gender-specific association is that of primary sclerosing cholangitis (PSC). Here, the male sex is clearly a risk factor [odds ratio (OR) up to 2.771, *p *= 0.022] *(* 30, 32). Likewise, in men, ankylosing spondylitis and the extremely rare amyloidosis are more common (31, 33), whereas, in women, joint, skin, and eye involvement seem to be more frequent (7, 16, 30, 34–36).

## Prognosis and disease activity

A very recently published study reports that there is a longer diagnostic delay in women than in men for both CD and UC due to a drawn-out evaluation of women, with a higher number of misdiagnoses at all levels of the healthcare system (37). It is well known that delayed diagnosis is a risk factor for the need for surgical intervention later in the course of the disease (38). Whether gender alone is a potential risk factor for greater disease activity and a more complicated disease course has been little studied and the results are contradictory. A population-based study by Romberg-Camps et al., in which the data sets of 1,187 patients were examined, could not find any evidence for gender as a prognostic factor for severe disease progression, but a longitudinal study from Israel, in which the data of 269 patients with Crohn’s disease were analyzed over a period of 10 years, identified male gender as a clear risk factor for a complicated course of the disease (OR 2.6, 95% CI: 1.17 to 5.75) (39). However, when looking at remission rates in relation to gender, a large cohort of IBD patients from the Rhine-Main region in Germany showed significantly higher remission rates in male UC patients (Crohn’s disease, 58.5% vs. 53.0%, *p*=n.s.; and ulcerative colitis, 69.4% vs. 58.0%, *p*=0.013) than in female UC patients (40). Another study of a German cohort of 1,032 patients with Crohn’s disease came to a similar conclusion; here, women with Crohn’s disease showed increased disease activity compared with men (41). In the inception cohort study iCREST-CD, male sex was significantly associated with the presence of perianal lesions (42).

## IBD-related complications

It is particularly interesting to look at the gender differences in the risk of osteoporosis. For example, although women with Crohn’s disease have an increased long-term risk of osteoporosis, in a large retrospective German study the proportion of men with osteopenia or osteoporosis (53.9%) was significantly higher than that of premenopausal women with IBD (29.6%). In this study, the risk is in the same range as in postmenopausal women with IBD (47%) (43, 44).

Male gender thus appears to be an independent risk factor for the development of bone density reduction, an observation that has been confirmed in several studies (16, 45, 46). This phenomenon is certainly underestimated in everyday clinical practice and requires increased attention, screening, and prevention. However, women and patients with stomas have also been shown in some studies to have an increased risk of osteoporosis, which also needs to be considered in daily clinical practice (47, 48).

Compared with the normal population, patients with IBD have an increased risk of colorectal cancer (CRC) regardless of gender (in men, RR 2.6, 95% CI: 2.2 to 3.1; in women, RR 1.9, 95% CI: 1.5 to 2.4) (49). However, there are also clearly gender-specific differences. Men with IBD have an increased risk of developing CRC compared with women with IBD (49, 50). Based on 171,000 person-years of follow-up, men have an approximately 60% increased risk compared with women (RR 1.6, 95% CI: 1.2 to 2.2). The cumulative incidence of CRC 40 years after initial diagnosis of IBD was 8.3% (men) vs. 3.5% (women) (49). The higher risk of CRC in male patients is not exclusive to patients with IBD; the same applies to the general population and can mostly be attributed to a number of risk factors. Male patients are more likely to smoke and consume more alcohol, processed meat, and red meat (51). The mortality of CRC is also higher in men with UC than in women with UC (the standardized mortality ratio (SMR) for men is 1.8, 95% CI: 1.0 to 3.1; for women it is 0.7, 95% CI: 0.3 to 2). In contrast, according to this meta-analysis, women have an increased mortality from pulmonary complications (in women, SMR CD 2.1, 95% CI: 1.4 to 3.0, vs. in men, SMR CD 1.5, 95% CI: 0.9 to 2.6; and, in women, SMR UC 1.6, 95% CI: 1.1 to 2.2, vs. in men, SMR CD 1.1, 95% CI: 0.6 to 2.1) (50).

## Drug therapy

For over a decade, gender-specific differences in response to therapy have been the focus of studies in other diseases. These include cardiovascular diseases (52, 53), rheumatoid diseases (54, 55), endocrinological disorders such as diabetes mellitus (56), and cancer (57). For IBD, however, these studies are mostly lacking and only isolated data secondary to studies conducted with a different primary end point are available. In the Dutch IBD Biobank study and the COIN study, no significant differences regarding the use of IBD-specific medication were observed, with the only exception being a more frequent use of prednisone in male CD patients than in female CD patents (6.8% vs. 3.7%, *p*=0.03) *(* 16). Much higher use of prednisone, also pronounced in men, was found in a large retrospective monocentric study (73.5% vs. 67.4%, *p*=0.04). The use of immunomodulators was also higher for men than women in this study (86.6% vs. 78.3%, *p*=0.008) *(* 58). It might be worth mentioning that young male EBV-negative patients treated with thiopurines, especially in combination with TNF-blocker therapy, display an increased risk of hepatosplenic lymphoma (59).

In a separate analysis, it was observed that 13.4% of women with Crohn’s disease, in contrast to 4.6% of men, did not receive IBD-specific drug therapy (*p*< 0.01) (40). This presumed undertreatment was also reflected in the medications used, with men being treated more frequently with immunosuppressive medications for both Crohn’s disease (men, 46.9%, vs. women, 37.9%) and ulcerative colitis (men, 34.3%, vs. women, 26.3%). This distribution was also found in the biologic therapy used for both Crohn’s disease (men, 6.9%, vs. women, 4.5%) and ulcerative colitis (men, 2.5%, vs. women, 1.4%). No correlation with the age or disease activity of the patients could be found in the examinations. In line with these data, the work of Lesuis et al. also demonstrated an increased use of biologics in men with IBD (54). It is noteworthy that this effect was also observed in patients with psoriasis and rheumatoid arthritis (54). Similarly, this work documented higher subjective, but not objective, disease scores in female patients with CD and rheumatoid arthritis. Based on these findings, justified concerns that too little attention is paid to gender differences in therapy management in IBD were raised (60). Several factors might contribute to the observed undertreatment in women. Many of our patients are in their reproductive years, and hesitance to use proper medication might be associated with the unfounded fear that medication can harm the fetus or impair fertility. For some women, the access to specialized care might be difficult, and this applies especially to patients who have migrated from other countries and do not have adequate health insurance and knowledge regarding the healthcare system.

## Treatment response

Predictors of response to a particular therapy have been repeatedly investigated; a systematic review of a total of 11 studies with 995 included CD patients treated with adalimumab identified male gender as an independent risk factor for loss of response and the need for dose escalation (58, 61). However, these data are also controversial, as gender was never the primary end point in the studies. In addition, a monocentric study (*n*=210 patients) is available that shows both a better and a longer response rate to therapy with infliximab in men with Crohn’s disease (62). In a smaller study in severe ulcerative colitis, where infliximab was used as a rescue therapy, female gender was a prognostic factor for higher remission rate at 1 year and higher cumulative non-colectomy rate (63, 64). In a very recently published individual-participant meta-analysis including data from clinical UC trials only, male patients with active UC receiving TNF-blockers had a significantly lower likelihood of clinical remission, and even mucosal healing, than women (65). In contrast, a small study with a median follow-up of 6 years revealed that drug survival was higher in males than females patients (48.1% vs. 30.8%, *p*=0.016) *(* 63). It can be argued here that staying on a drug alone does not prove its efficacy on end points such as clinical remission or even mucosal healing.

The reasons for the abovementioned differences remain speculative and are not well explained by the available data. One possible explanation is offered by the work of Zelinkova et al., who demonstrated a significantly higher adverse event rate in women in a retrospective analysis of 1,009 patients. Reduced medication use by female patients with IBD (66). Another study was able to confirm this for adalimumab; this cohort included a total of 107 women and 81 men; here, too, there were significantly more adverse drug reactions in the female IBD patients, which also led more frequently to the termination of therapy (63). In a retrospective study of 529 IBD patients treated with TNF-blockers, female sex was associated with drug discontinuation due to side effects (HR 4.05, 95% CI: 2.36 to 6.98) (67).

## Therapy adherence

In all chronic diseases, adherence to therapy is of great importance in strategic considerations, as it is the only way to ensure effective remission-maintaining therapy. Therapy adherence is therefore as problematic in IBD patients as in patients with other chronic diseases (68–70). That it is a key issue is shown by an internet-based cohort from North America, in which 41% of IBD patients self-reported poor adherence (69). In this study, low inflammatory activity correlated with high levels of adherence. The lack of adherence affects both sexes, with older studies showing reduced adherence in men with IBD (68, 70), and more recent work showing that it is more likely to be reduced in young women (71–73). Especially in the setting of a therapy break, it should be noted that men have a higher probability of relapsing.

## Surgical therapy

There have been retrospective cohort studies that have shown different gender-specific risk constellations. However, these data cannot be confirmed in more recent analyses, and nowadays much attention is paid to the prevention of postoperative recurrence of Crohn’s disease, and drug therapy is often initiated postoperatively (74).

However, some gender-specific risk factors have been identified. For example, in the Olmsted County cohort, male sex was identified as a risk factor for major abdominal surgery (hazard ratio (HR) 1.6) (75). Another retrospective analysis showed a higher rate of ileocecal resections in women (44% vs. 32%, *p*=0.004) *(* 7). This study also reported that women had an increased risk of a second resection.

For ulcerative colitis, reference can be made to two population-based studies that identified a higher rate of colectomy in men (HR 2.1, 95% CI: 1.3 to 3.5; and HR 2.6, 95% CI: 1.58 to 4.36) (76, 77). Pouch-specific complications such as ileus, fistulae, and pouch failure appear to be more common in women (78). Men, on the other hand, are more likely to develop antibiotic-refractory pouchitis (16.3% vs. 10.7%, *p*=0.001) *(* 79).

## Psychosocial factors

The most consistent gender differences in IBD relate to psychosocial functioning. Although depression, anxiety disorders, eating disorders, and sexual dysfunction also occur in male IBD patients, these are described far more frequently in women (80), an observation that can also be made in the general population [McLean CP, Asnaani A, Litz BT, Hofmann SG. Gender differences in anxiety disorders: prevalence, course of illness, comorbidity and burden of illness. J Psychiatr Res. 2011 Aug;45(8):1027–35. doi: 10.1016/j.jpsychires.2011.03.006. Epub 2011 Mar 25. PMID: 21439576; PMCID: PMC3135672.]. The first problem is that these partly psychological, partly psychiatric problems are underdiagnosed and undertreated, especially in female IBD patients (80, 81). In a recently published meta-analysis that included 77 studies representing over 30,000 IBD patients, women were more likely to have symptoms of anxiety than men (for women, pooled prevalence 33.8%, 95% CI: 26.5 to 41.5, vs. for men, 22.8%, 95% CI: 18.7 to 27.2; OR 1.7, 95% CI: 1.2 to 2.3). They were also more likely to have symptoms of depression than men (for women, pooled prevalence 21.2%, 95% CI: 15.4 to 27.6, vs. for men, 16.2%, 95% CI: 12.6 to 20.3; OR 1.3, 95% CI 1.0 to 1.8) (82). The authors conclude that encouraging gastroenterologists to screen for and treat these disorders might improve outcomes for patients with IBD. For example, screening for depression is supported by the recommendations provided by the United States Preventive Task Force (PSPSTF) universal screening in all adult populations, including pregnant and postpartum women, and the American College of Gastroenterology (AGA) screen for depression and anxiety in patients with IBD (83, 84).

**Figure 1:**
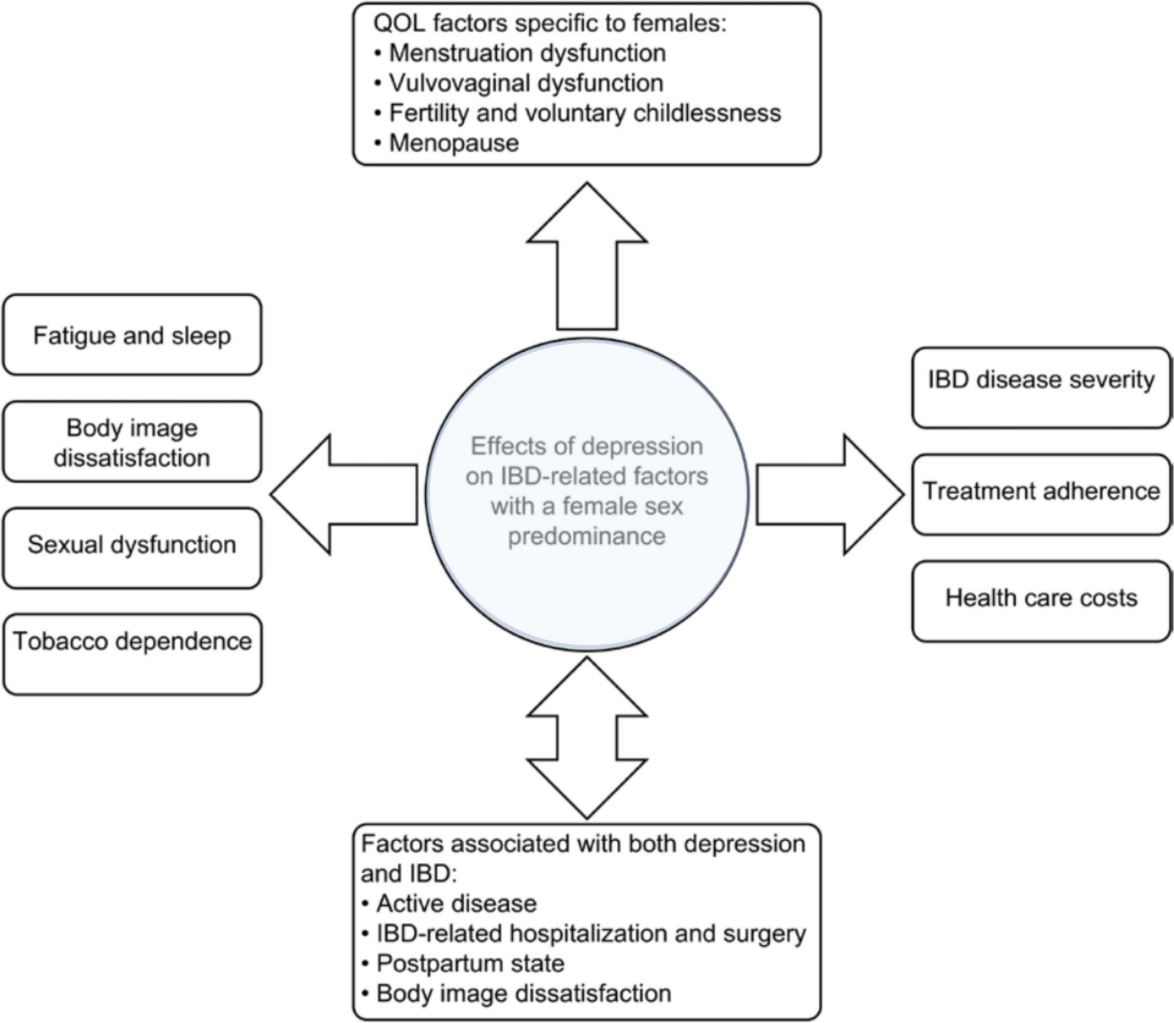
Factors associated with female sex and depression in patients with IBD. (adapted from Bommena et al. (80))

Bommena et al. have described the effects of depression on IBD-related factors, with the female sex predominant in a recently published review (see **Figure 1**). The consequences of depression, especially in female IBD patients, are discussed in detail. Only a few should be mentioned here: women with IBD are more likely to experience postpartum depression than women without IBD (85). Over 50% of women with IBD are suffering from sexual dysfunction (SD) compared with 28% in healthy controls (*p*<0.01), vs. 16.9% in men with IBD compared with 7.4% in healthy controls (p=0.64) (86). Approximately 70% of women with IBD are concerned about their body image (87, 88), a concern that significantly impairs their quality of life (QOL), which is already reduced in female IBD patients (89). Another factor contributing to poor QOL in women with IBD is fatigue. In the Swiss IBD cohort, 672 IBD patients (55.6%) reported significant fatigue compared with 145 (35%) controls (OR 2.71, 95% CI: 2.08 to 3.54; *p*< 0.001). In IBD, fatigue also significantly affected the daily activities (FSS ≥ 4) of 405 (33.5%) IBD patients vs. 81 (19.6%) controls; *p*< 0.001). In the MANOVA model, fatigue levels were associated with female gender (coefficient 0.839, 95% CI: 0.556 to 1.123; *p*< 0.001), younger age at diagnosis (−0.031 per year, 95% CI: −0.042 to −0.019; *p*< 0.001), shorter disease duration (−0.036 per year, 95% CI: −0.050 to −0.022; *p*< 0.001), nocturnal diarrhea (0.718, 95% CI: 0.295 to 1.141; *p* = 0.001), low educational level (*p* = 0.034), and symptoms of depression and anxiety (90).

Depression in male IBD patients is less prevalent; however, it also affects their QOL. Depression is related to SD, as mentioned above, which is prevalent in 10%–40% of male patients with IBD (91). Risk factors for male SD in IBD are advanced age, severe disease, post-IBD-related surgery, and smoking (91, 92). Men experience fear of infertility and body image dissatisfaction, which is also strongly correlated to depression and subsequently impairs their QOL (93–95).

Most importantly, a multimodal integrative therapeutic approach is feasible, has been shown to be successful in both male and female IBD patients, and should be implemented more widely (80).

## Pregnancy and breastfeeding

Since IBD is often diagnosed in young adults family planning should be considered throughout the treatment. Here the male perspective on fertility and family planning is much less illuminated than the female view (95, 96). Patients with IBD have fewer children than people without an IBD diagnosis. Interestingly, actual reduced fertility due to the disease does not seem to be the cause; rather, it is mostly unfounded, diffuse fears that play the biggest role. In both sexes, there is great uncertainty about the risk of passing on the disease to the child (97). In female patients, the fear of possible complications during pregnancy due to the disease or drug therapy during pregnancy also plays a major role in self-chosen childlessness (98). Another aspect of family planning affecting both sexes is adequate nutrition with a balanced vitamin and mineral content. Not only can malnutrition lead to reduced fertility in men and women (94), but a folic acid deficiency in women can lead to neural tube defects in the embryo. The fact that early comprehensive counseling on the topic of childbearing and pregnancy leads to a better course of pregnancy and healthier newborns has been well studied for women (99). These studies are not yet available for men .

Turning to the aspects of drug therapy in family planning and pregnancy, even greater differences between the sexes are obvious. In one study it was shown that for men a preserved remission of the disease in the procreation phase is of great importance. There is hardly any concern that taking medication could be a problem here or could limit male fertility (100). However, it has been repeatedly shown that this plays a much greater role for woman, especially if they have not received adequate counseling beforehand. Most patients are convinced that drug therapy at the time of conception and further course of pregnancy is significantly more problematic for the unborn child than an increase in disease activity when the previous therapy is discontinued (101, 102).

## Summary and outlook

**Figure 2:**
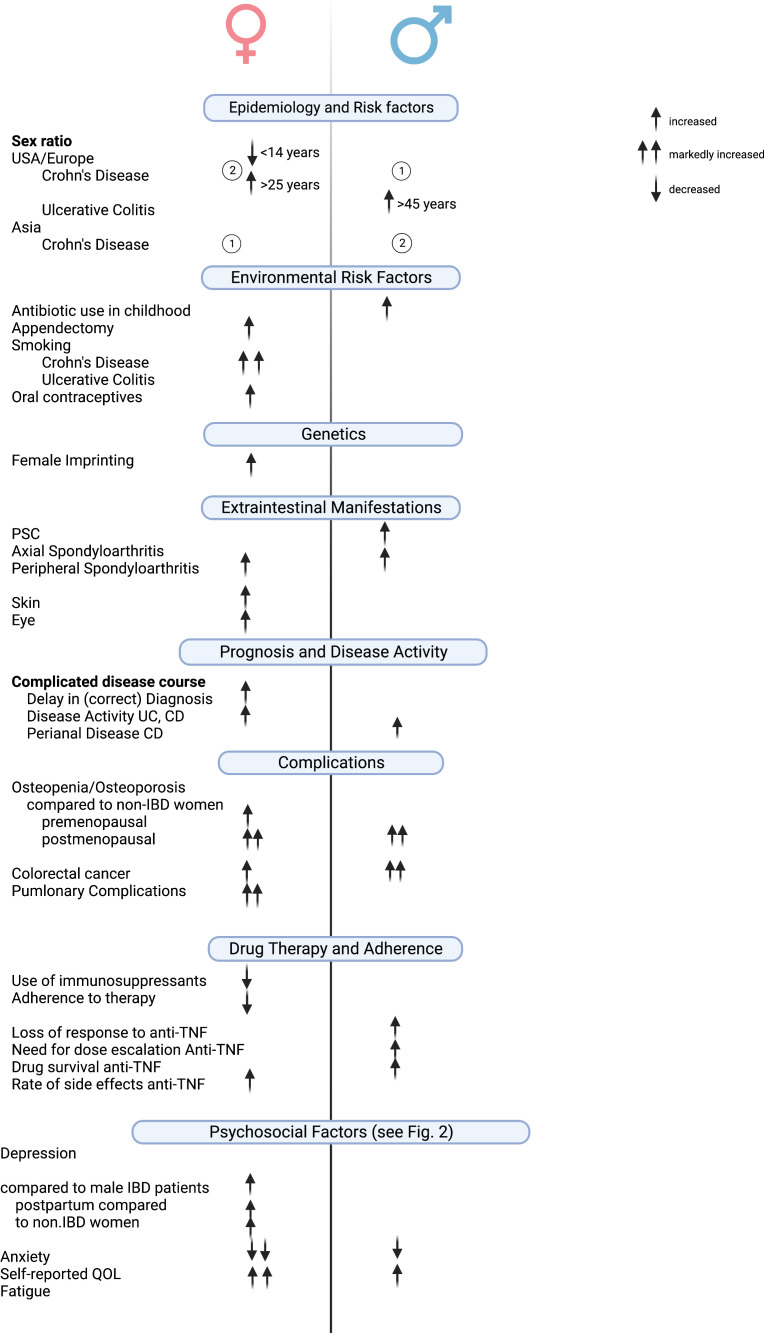
Clinically relevant gender/sex-based differences in IBD. Created with Biorender.com.

This review provides an up-to-date overview of sex- and gender-specific aspects of IBD in terms of disease phenotype, disease course, and treatment outcome (**Figure 2**). It should be noted that most of the differences described are based on retrospective data or from studies that did not have the primary end point of identifying these differences.

The most significant and clinically meaningful sex- and gender-specific differences in IBD relate to psychosocial functioning. Although depression, fatigue, anxiety disorders, eating disorders, and sexual dysfunction also occur in male IBD patients, women seem to be affected much more frequently and severely in these areas.

It seems important that, analogous to other chronic diseases, studies with sex- and gender-specific end points are conducted in the future to enable specialized, personalized medical care for patients with IBD.

## Author contributions

IB and ES drafted the manuscript. All authors contributed to the article and approved the submitted version.
